# Design of a Novel Telerehabilitation System with a Force-Sensing Mechanism

**DOI:** 10.3390/s150511511

**Published:** 2015-05-19

**Authors:** Songyuan Zhang, Shuxiang Guo, Baofeng Gao, Hideyuki Hirata, Hidenori Ishihara

**Affiliations:** 1 Graduate School of Engineering, Kagawa University, 2217-20 Hayashi-cho, Takamatsu, Kagawa 761-0396, Japan; 2 The Institute of Advanced Biomedical Engineering System, School of Life Science and Technology, Key Laboratory of Convergence Medical Engineering System and Healthcare Technology, The Ministry of Industry and Information Technology, Beijing Institute of Technology, Haidian District, Beijing 100081, China; E-Mails: guo@eng.kagawa-u.ac.jp (S.G.); gaobaofeng@bit.edu.cn (B.G.); 3 Department of Intelligent Mechanical Systems Engineering, Kagawa University, Kagawa 761-0396, Japan; E-Mails: hhirata@eng.kagawa-u.ac.jp (H.H.); ishihara@eng.kagawa-u.ac.jp (H.I.)

**Keywords:** telerehabilitation system, closed-loop interaction control strategy, series elastic actuator, force-sensing mechanism, inertia sensor, contact-less angle sensor, force sensor

## Abstract

Many stroke patients are expected to rehabilitate at home, which limits their access to proper rehabilitation equipment, treatment, or assessment by therapists. We have developed a novel telerehabilitation system that incorporates a human-upper-limb-like device and an exoskeleton device. The system is designed to provide the feeling of real therapist–patient contact via telerehabilitation. We applied the principle of a series elastic actuator to both the master and slave devices. On the master side, the therapist can operate the device in a rehabilitation center. When performing passive training, the master device can detect the therapist’s motion while controlling the deflection of elastic elements to near-zero, and the patient can receive the motion via the exoskeleton device. When performing active training, the design of the force-sensing mechanism in the master device can detect the assisting force added by the therapist. The force-sensing mechanism also allows force detection with an angle sensor. Patients’ safety is guaranteed by monitoring the motor’s current from the exoskeleton device. To compensate for any possible time delay or data loss, a torque-limiter mechanism was also designed in the exoskeleton device for patients’ safety. Finally, we successfully performed a system performance test for passive training with transmission control protocol/internet protocol communication.

## 1. Introduction

In the United States, approximately 795,000 new or recurrent strokes are reported annually [1]. Strokes can lead to the impaired motor control of the upper and lower limbs with significant impairment of activities of daily living (ADL). Upper-limb function is the most important requirement of many ADL. Improving upper-limb ability after a brain lesion requires early and intensive therapy [2]. An effective and stable rehabilitation process can be offered by robot-assisted therapy [3]. Most existing therapeutic robots fit into one of these categories: endpoint manipulator [4,5], cable suspension [6], and powered exoskeleton [7,8,9]. However, the vast majority of rehabilitation robots cannot be moved out of rehabilitation centers and require supervised assistance from qualified personnel [10]. Considering the inconvenience of regularly attending these centers, patient demand for home-based rehabilitation is expected to increase in the future. Although home-based rehabilitation devices have been developed [11], caregivers usually lack the skills to operate them in a professional manner; therefore, telerehabilitation systems are a logical next step. Such a system would be installed in the patients’ residence, enabling therapists to remotely treat or assess the patient instead of visiting the patient’s house, and avoiding the need for patients to attend a clinic. Therapists could treat their patients at fixed times, and accordingly plan their duties. For example, the Georgetown University Imaging Science and Information Systems Center has already assembled two InMotion2 robots into a telerehabilitation test bed. The therapist and patient robots can independently interact with a virtual object. The virtual object senses and calculates a force that is transmitted to both the therapist and patient [12]. The telerehabilitation system developed by Holden *et al.* [13] allows a remotely located therapist to administer treatment through a virtual-environment-based motor training system. The patient’s movements are animated with a virtual scene, which the therapist can direct and monitor in real time. However, studies of robot-assisted stroke rehabilitation have shown that robots can outperform humans in mundane care but not in intensive manual therapy [14]. Therefore, the loss of real contact feeling between the therapist and patient remains to be a problem in telerehabilitation. Some studies have attempted to provide a real contact feeling between the therapist and patient in telerehabilitation. For example, a portable telerehabilitation system designed by Park *et al.* [15] can detect the elbow’s flexion/extension angle and torque at both the master and slave devices. The system allowed the therapist to remotely evaluate the impaired elbow of stroke survivors by operating a mannequin arm driven by a master device. To minimize the effect of network latency, a real-time control strategy and a teach-and-replay control method are used for tasks involving slow movements and fast movements, respectively. To overcome the loss of transparency and the instability of telerehabilitation systems caused by time delays, some other control strategies have also been designed [16,17]. 

The novelties of our designed system with regard to existing telerehabilitation systems are summarized as follows: first, we aimed at designing a low-cost master device having a compact structure. Therefore, we abandoned the expensive force sensor and opted instead to use a motor with a large reduction ratio gearhead. However, the master device used by therapists should provide variable impedances or detect the extra force applied on the master device by the therapist when switching between passive and active training. The difficulty was solved by applying a series elastic actuator (SEA) that can provide variable impedances and resolve the non-backdrivability problem caused by the large reduction ratio gearhead. The force-sensing mechanism, which is executed during active training, in SEA operates in a manner similar to a force sensor. Second, we designed a compliant exoskeleton device for patients. The mechanism design resolved the joint misalignment between the robot and human joint. A torque-limiter mechanism is incorporated in the exoskeleton device to protect the patient from excessive torque. Most of existing therapeutic devices, especially the exoskeleton type are bulky and expensive. However, the SEA makes the totally weight 1.3 kg while guaranteeing sufficient force/torque performance. Further, the active training can be performed as the output impedance of the device can be adjusted to as low as near-zero [11]. Third, the telerehabilitation system has the potential to provide a real contact feeling for both passive and active trainings. 

The remainder of this paper is divided into four parts. In Section 2, we introduce the overview of the proposed system. We describe in detail the procedures utilized by the telerehabilitation system in passive and active training with a real contact feeling. In Section 3, we present the mechanism design and control method of both the master and slave devices. The safe execution part in the exoskeleton device is introduced in this paper. The human-upper-limb-like device (master side) will be introduced in detail. We present the experiments and results in Section 4. The discussion and conclusions are provided at the end. 

## 2. Overview of the Proposed Telerehabilitation System

### 2.1. Conceptual Design of the Telerehabilitation System

**Figure 1:**
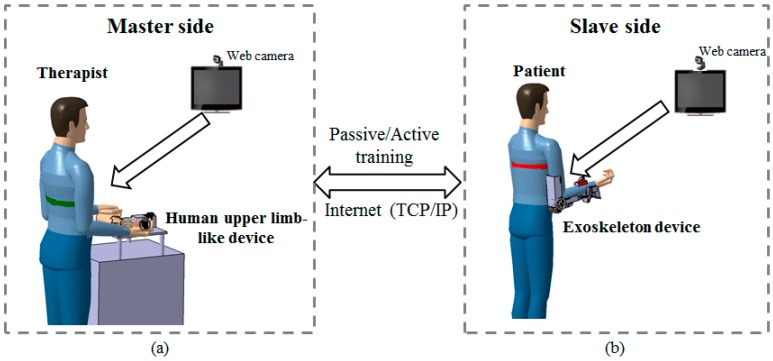
(**a**) Master side located at the rehabilitation center; (**b**) Slave side located in the patient’s house.

We designed the proposed system to meet the requirements of telerehabilitation with therapists’ sensory input. The conceptual design is presented in Figure 1. On the master side (Figure 1a), the therapist operates a human-upper-limb-like device in a rehabilitation center while monitoring the status of the patient with a web camera. The therapist’s sensory information input to the master device is transmitted to the slave side and detected by the patient via the exoskeleton device (Figure 1b). The master device can also track the patient’s motion for active training, in which the patient’s motion is dominant. For ensuring patient safety, the exoskeleton device (slave side) can detect spasms from the motor’s current. A spasm is defined as an involuntary contraction of a muscle or a group of muscles [18]. The master device is designed with a SEA, and the output impedance can be adjusted by controlling the deflection of the elastic elements. Therefore, in the case of a spasm, it is possible to increase the output impedance of the master device; thus, therapists can react quickly to decrease the interaction force/torque. The force-sensing mechanism applied in the master device can also detect the extra force that the therapist adds for accomplishing active training. Because of a possible time delay or data loss during telecommunication, the exoskeleton itself also has a torque-limiter mechanism. As soon as an interaction torque beyond the predefined threshold is applied from or to the axis, the torque-limiter mechanism can force the device to rotate freely regardless of the position of the motors.

### 2.2. Passive and Active Training Accomplished by the Proposed Telerehabilitation System

Passive exercise is suitable for patients with severe impairments and poor motor function [19]. During the initial stage of the rehabilitation process, such patients typically cannot move autonomously; therefore, training should provide a desired motion pattern under therapist supervision. In a one-on-one situation, the therapist can directly assist the patient in performing tasks. The force exerted and the operational skill depends on the therapist’s subjective adjustment and experience. However, if the therapist and the patient are physically separated, the therapist cannot directly sense the patient’s condition. 

**Figure 2:**
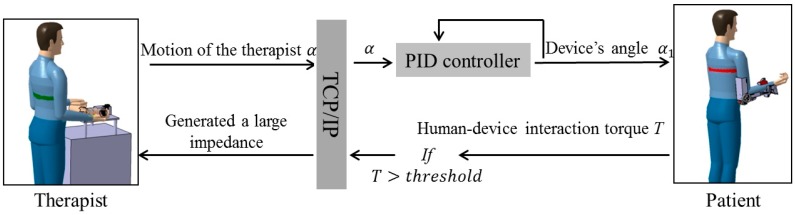
Schematic of passive training.

Figure 2 is a schematic depicting passive training with the telerehabilitation system. It is a solution to the difficulties associated with teleoperation. On the therapist’s side, a high weight-to-torque ratio actuator has been applied to the master device to guarantee sufficient force/impedance performance for therapists. The subsequent non-backdrivable problem was solved by applying the SEA mechanism. By controlling the deflection of the elastic elements to near-zero, the therapists can operate the device freely regardless of the non-backdrivable property. During special situations, e.g., in the case of spasms, the deflection of the elastic elements can also be controlled to output enough impedance. First, the motion of the therapist α can be detected with the inertia sensor (MTx sensor, Xsens, Enschede, The Netherlands) installed on master device by setting the deflection of elastics to near-zero, and then α is transmitted to the relaxed patient located at the patient’s house. Tracking accuracy is ensured by a closed-loop position control and α_1_ is the rotation angle of the slave device. If the patient experiences pain or an involuntary contraction of a muscle during the exercise, the torque *T* calculated from the actuator’s current *I_m_* will increase. Further, the relationship between torque *T* and motor current *I_m_* can be calculated with Equation (1), where *l* (a coefficient of motor) equals to 23.5 mNm/A, and n is the ratio of the gearhead [20]. If *T* exceeds the therapist-specified threshold, it can be fed back to the therapist through the master device. The torque-limiter mechanism incorporated in the exoskeleton device can also release the motor shaft if the therapist does not react in a timely fashion to decrease the interaction force/torque or there is a loss or delay of data during telecommunication. The therapist can also monitor patient status better by using the web camera.

(1) $$T=nlI_{m}$$

**Figure 3:**
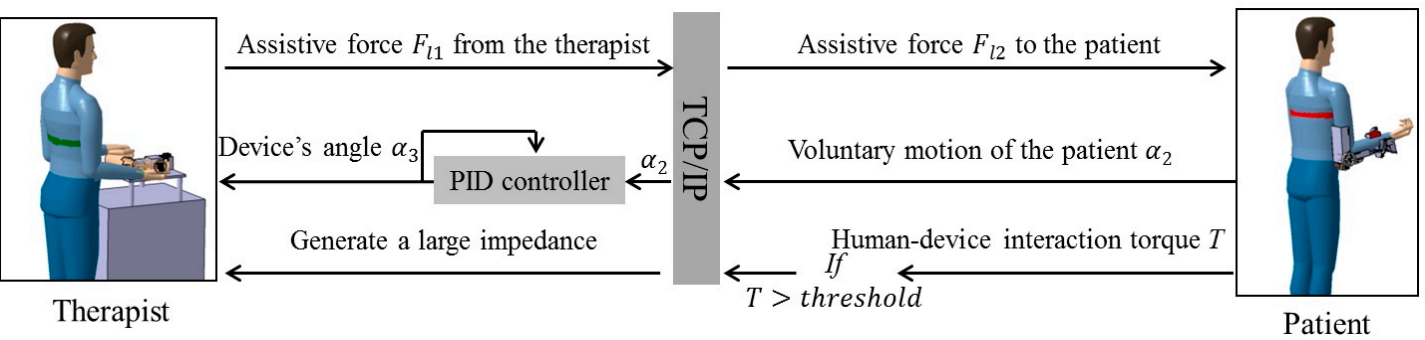
Schematic of active training.

On the other hand, active training, which is suitable for weak patients with limited motor function [21], can also be performed with the proposed telerehabilitation system. The main difficulty was the design of the exoskeleton device (slave side). The difficulty was caused by applying a high weight-to-torque ratio actuator. The actuator successfully decreased the weight of the total device to 1.3 kg while guaranteeing sufficient force/torque performance to execute the rehabilitation tasks. However, building both active and passive modalities into this exoskeleton device became generally difficult because the modalities have different functional requirements. In contrast to the passive modality, which requires a relatively high joint impedance of the exoskeleton device, the active modality requires a near-zero impedance [9]. To obtain variable impedances, the device was designed with elastic elements (elastic belts) in order not to be rigid. Therefore, patient–device synchronization can be achieved by tracking the motion of patient’s limb with an inertia sensor (MTx sensor) and then controlling the deflection between the exoskeleton device and the patient’s limb [20]. The control method can also be described as a closed-loop interaction control strategy [22]. Figure 3 is a schematic depicting active training with the telerehabilitation system.

Relatively weak patients with limited motor function are suitable to participate in active training, where the patient’s voluntary motion is dominant. Once the patient is not able to achieve tasks, the exoskeleton device will provide partial assistive force/torque. The therapist on the remote side can determine the level of assistive force necessary, as shown in Figure 3. In more detail, the voluntary motion (angle of elbow flexion/extension) α_2_ from patients is detected with an inertia sensor (MTx sensor) that is fixed on the forearm. The user–device interaction force of the exoskeleton device should be set to near-zero so that the patient can voluntarily move his/her arm. This is accomplished by controlling the exoskeleton device while synchronously tracking the motion of the patients. After α_2_ is transmitted to the master side, the master device tracks the motion of the patients. The tracking accuracy is ensured with a closed-loop position control, and α_3_ is the rotation angle of the master device. The therapist can add assistive force *F_l_*_1_ to the master device; *i.e.*, the therapist can treat the master device as if it were the patient’s limb. Owing to the benefits of the SEA adopted by both the master and slave devices, the force control is converted to a position control following the Equation (2), where coefficients *k*_1_ and *k*_2_ relate the deflection of the elastic elements to the sensed load force. The safe execution for patients is same with passive training by measuring the motor’s current from the exoskeleton device and using the torque-limiter mechanism:

(2) $$F_{l1}/k_{1}=F_{l2}/k_{2}$$

## 3. Mechanism Design and Control Method 

### 3.1. Mechanism Design and Method of Control for the Master Device 

**Figure 4:**
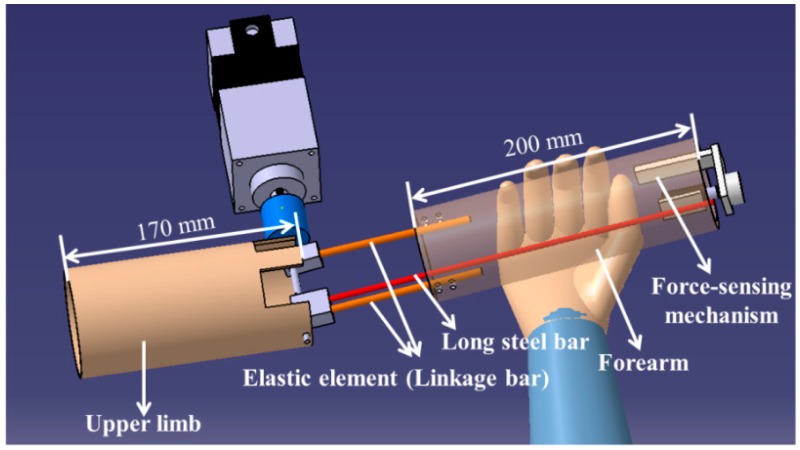
Prototype of the master device (*i.e.*, a human-upper-limb-like device).

A prototype of the master device was designed as shown in Figure 4. As mentioned above, we selected a weight-to-torque ratio motor (ARM66AC-PS25; Oriental Motor, Tokyo, Japan) to provide sufficient force/torque performance. The maximum nominal output torque of the driving unit is limited to 16 N·m by the mechanical strength of the planetary gear. An unavoidable problem with large reduction ratio gearheads is non-backdrivability resulting from the high-reflected inertia and friction; *i.e.*, the motor cannot be turned by an outside force acting on its output shaft. To obtain variable impedances or a compliant joint, elastic elements have to be added between the motor shaft and output of the device. This kind of mechanism is called an SEA, and it can provide many benefits in force control [23,24,25]. Specifically, SEAs realize high shock tolerance, low reflected inertia, high energy-storage capacity, and accurate/stable force control. The newly proposed design in the master device measures the elastic element deflection, and the deflection is relative to the sensed force. To magnify the deflection and measure it at the extreme part of the master device, a long steel bar is installed on the motor shaft parallel to the elastic element. This method can effectively decrease the signal-to-noise ratio, because the deflection of elastic elements is usually designed quite small to enlarge the force bandwidth.

**Figure 5:**
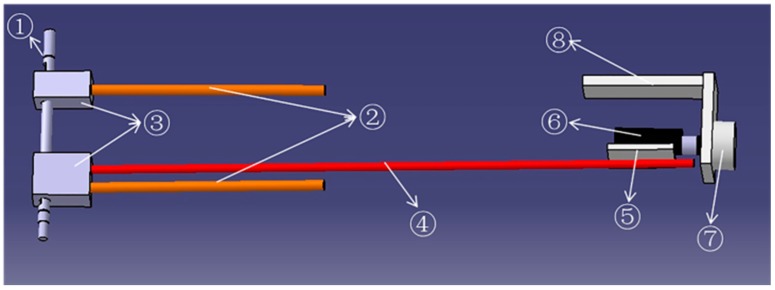
The force-sensing mechanism. (① a steel shaft connects to the motor shaft; ② two steel bars act as the elastic element; ③ connectors; ④ long steel bar; ⑤ a rubber sheet connected to the long steel bar; ⑥ connector; ⑦ angle sensor; ⑧ connector).

Detailed information on the force-sensing mechanism we installed in the master device is shown in Figure 5. We used the contactless Hall-IC angle sensor (CP-20HB; Midori Precisions Co., Ltd., Tokyo, Japan) to measure the deflection. This sensor contains a metal sleeve bearing with absolute linearity (±1% force sensing). The force-sensing mechanism is a sensor system for measuring the deflection of the elastic elements. However, the master device can output variable impedances by controlling the deflection of the elastic elements [26,27]. Specifically, the output impedances of the master device can suddenly increase when the patient experiences pain or spasms. During active training, the force-sensing mechanism can also measure the extra force applied to assist a patient’s motion. As shown in Figure 5, part ⑥ is fixed to the rotation shaft of the angle sensor, while part ⑤is fixed to the long bar. Therefore, the deflection of the elastic elements is related to the rotation angle θ of the angle sensor and the deflection can be calculated with Equation (3), where r is the radius of part ⑥, and *L*_3_ and *L*_4_ represent the length of the elastic elements and the long steel bar, respectively:

(3) $$L_{1}=\frac{\theta \pi r}{180}\cdot \frac{L_{3}}{L_{4}}$$

The control system used for the master system comprises two parts: a high-level control system (Windows 7 Professional system with a 3.0-GHz AMD Processor and 4.0-GB random-access memory) and a low-level actuator control system (DSP2812 processor inside controller). The two systems can communicate by a serial port. A user interface in the high-level control system was programmed in Visual C++ 2010 (Microsoft Co., Redmond, WA, USA). The user interface provides several types of useful data to the therapist, including real-time video from the web camera and some patient statistical data, e.g., motion and assisting force. An angle sensor and an inertia sensor are used in the master system. The inertia sensor (MTx sensor) is installed on the forearm part of the master device to measure the rotation angle of the master device. The function of the angle sensor has been mentioned above. The MTx sensor combines a triaxial accelerometer, a triaxial gyroscope, and a triaxial magnetometer. By using a sensor fusion algorithm, the MTx sensor can output an accurate acceleration or angle [28].

A closed-loop interaction control strategy [25] is applied to the master device. With the control method, the output impedance of the master device can be controlled by adjusting the deflection of the elastic elements. In our designed telerehabilitation system, the rotation angle of the master device α and the deflection of the elastic elements *L*_1_ are the inputs of the controller. During operation, the load force *F_l_* imposes a deflection *L*_1_ on the elastic elements as follows:

(4) $$L_{1}=\frac{F_{l}}{K_{s}}$$

From Equation (4), the desired rotation angle of the motor *X_md_* with respect to the desired rotation angle of the master device *a* is computed as follows:

(5) $$X_{md}=a+\frac{F_{l}}{K_{s}}$$

Adding the virtual impedance model to the controller, we obtain the following:

(6) $$F_{l}=K_{v}a+B_{v}\dot{a}$$

where *K_v_* and *B_v_* are the desired coefficients for the virtual spring and damper model. By replacing Equation (6) with Equation (5), Equation (7) is obtained as follows:

(7) $$X_{md}-a=\frac{K_{v}a+B_{v}\dot{a}}{K_{s}}$$

Therefore, the generated virtual impedance of the master device can be controlled with Equation (7). A typical proportional–integral–derivative (PID) algorithm is written as Equation (8). The *e*(*t*) used in the master control system can be written as Equation (9), in which *L*_1_(*t*) is measured with the force-sensing mechanism. Therefore, by combining Equations (8) and (9), Equation (10) can be obtained, where *v*(*t*) is the control input for the motor:

(8) $$v(t)=K_{p}e(t)+K_{i}\int_{0}^{t}e(t)dt+K_{d}\frac{d}{dt}e(t)$$

(9) $$e(t)=(X_{md}(t)-a(t))-L_{1}(t)$$

(10) $$v(t)=K_{p}(X_{md}(t)-a(t)-L_{1}(t))+K_{i}\int_{0}^{t}\left(X_{md}(t)-a(t)-L_{1}(t)\right)dt+K_{d}\frac{d}{dt}(X_{md}(t)-a(t)-L_{1}(t))$$

We experimentally determined the parameters for the PID controller:

### 3.2. Torque-Limiter Mechanism Applied in the Exoskeleton Device (Slave Side) 

**Figure 6:**
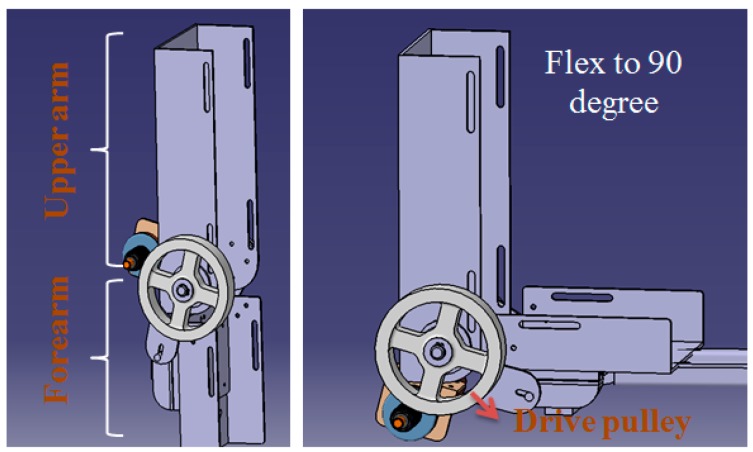
Design of the exoskeleton device (elbow joint).

The exoskeleton device applied on the slave side of telerehabilitation system is shown in Figure 6. The exoskeleton device (slave side) provides an ergonomic physical human–robot interface that is convenient to wear and comfortable to operate. The device is easily worn by caregivers or patients themselves. During movement, two passive degree of freedom (DoF) mechanisms in the elbow joint allow constant alignment between the user’s elbow and robot axis. Otherwise, joint misalignment between the robot and human joints would introduce unwanted translational forces. In the actuated DoF of the elbow joint, the power derived from the motor is transmitted to a drive pulley through a stainless steel cable. A similar structure can also be found in [18]. The detailed mechanism and control method of our exoskeleton device can be found in [11]. 

**Figure 7:**
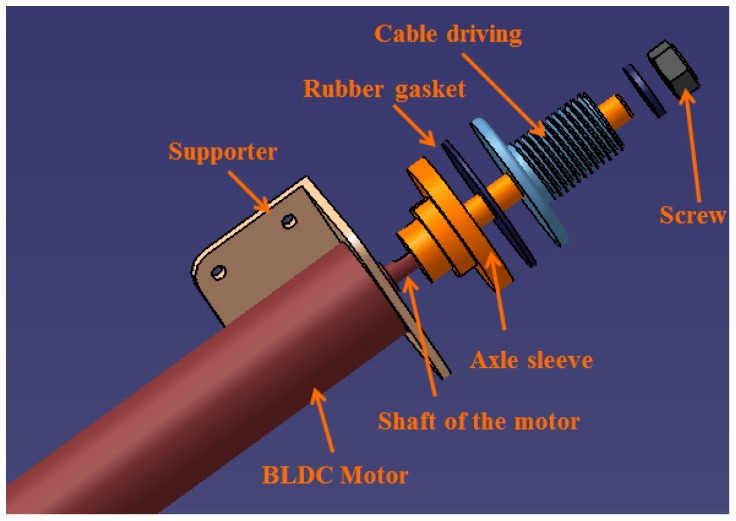
Torque-limiter mechanism in the elbow joint.

As mentioned in Section 2, safe execution for patients is guaranteed by measuring the motor’s current of the exoskeleton device and the torque-limiter mechanism. In a study by Oh *et al.* [29], a torque-limiter mechanism was also applied for knee rehabilitation. The torque-limiter mechanism can be released by excessive torque to protect the patient’s safety. The torque-limiter mechanism in our exoskeleton device is shown in Figure 7. The axle sleeve is fixed to the motor shaft. The cable driving part is connected to the axle sleeve by friction and the rubber gasket provides the friction force. The compression force between the axle sleeve and cable driving part can be adjusted with a screw; *i.e.*, the torque threshold of the torque-limiter mechanism can be adjusted by a screw. When external torque to the cable driving part is less than the threshold, the cable driving part can rotate together with the axle sleeve; *i.e.*, the motor can drive the device to move. If the external torque is larger than the threshold, the friction force can no longer connect the cable driving part and axle sleeve part together. Therefore, the cable driving part will rotate independent of the motor; thus, patient safety can be guaranteed. 

## 4. Experiments and Results

### 4.1. Calibration of the Force-Sensing Mechanism in the Master Device with the Force Sensor 

As mentioned above, an angle sensor is used for measuring the deflection of the elastic elements in the master device. It is necessary to calibrate the force-sensing mechanism before applying the closed-loop interaction control strategy. The purpose is to obtain a relationship between the load force *F*_l_ and the rotation of the angle sensor θ as well as the deflection of elastic elements *L*_1_. By combining Equations (3) and (4), Equations (11) and (12) are identified. Table 1 shows three parameters in Equation (12); therefore, *K* is a constant value:

(11) $$F_{l}=k_{s}\cdot \theta \cdot K$$

(12) $$K=\frac{\pi r}{180}\frac{L_{3}}{L_{4}}$$

**Table 1:** Parameters for the force-sensing mechanism in the master device.

Parameters	Value
Radius of Part ⑥ (*r*)	5 mm
Length of elastic elements (*L*_3_)	80 mm
Length of long steel bar (*L*_4_)	270 mm

**Figure 8:**
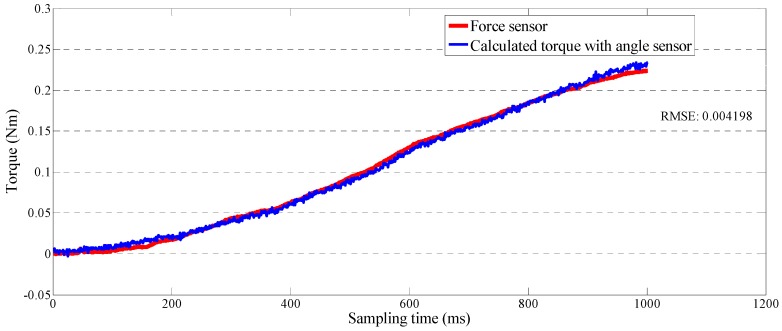
Calibration results for the force-sensing mechanism.

Without applying any control methods, the master device becomes non-backdrivable after motor excitation; *i.e.*, when a force is added on the master device, the motor will not rotate. Therefore, the added force will deflect the elastic elements. We installed a force sensor (FS03 force sensor; Honeywell, Morristown, NJ, USA) on the front end of the forearm part to perform the calibration. The force sensor is a peizoresistive-based force sensor. The sensor features a laser-trimmed thick ceramic film and an integrated circuit sensor element in small plastic housing. The extremely small size (25.1 × 17.27 × 8.26 mm) and light weight (approximately 3.5 g) was suitable to calibrate our master device. We recorded data from two sensors with a synchronous analog-to-digital board at a 1000-Hz sampling frequency. We performed the curve fitting with MATLAB (MathWorks Co., Natick, MA, USA). We used the root-mean-square error (RMSE) to evaluate the obtained fitting results. The calibration result is shown in Figure 8, where RMSE is 0.004198, and the sensed load *F_l_* can be varied with torque as shown in Equation (13) where *l_m_* is equal to 165 mm. The relationship between the data from the angle sensor and the force sensor is described by Equations (14) which was calculated with a curve-fitting method (linear polynomial):

(13) $$\tau_{l}=k_{s}\cdot \theta \cdot K\cdot l_{m}$$

(14) $$\tau_{l}=0.3578\cdot \theta +0.001652$$

### 4.2. Performance Test of the Output Impedances of the Master Device

**Figure 9:**
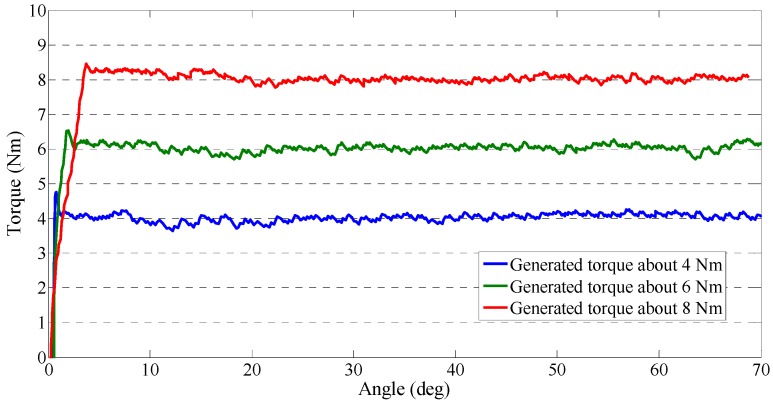
Constant torque from the master device.

**Figure 10:**
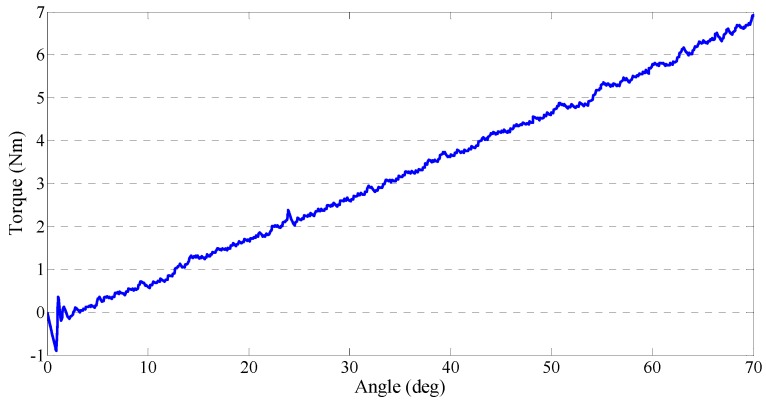
Gradually increased torque from the master device.

We introduced the closed-loop interaction control method for the master device in Section 3. We performed two experiments to test the performance of the output impedances. This performance may influence the stability of telerehabilitation in two aspects. First, in passive training, the deflection of the elastic elements is controlled as a near-zero value, so that the therapists can operate the master device freely without the non-backdrivable influence. Second, the master device needs to increase the output impedance in the case of patient spasm. Our experimental results are shown in Figure 9 and Figure 10. During the experiments, we measured force with the same force sensor (FS03 force sensor) used during data calibration, and we measured the rotation angle of the master device with an inertia sensor (MTx sensor). The generated torque can be adjusted from near-zero to about 16 Nm. In this experiment, three values of generated torque were selected to test the device’s ability to generate a constant torque. Figure 9 shows that the master device could generate a stably constant torque regardless of the rotation of device. The time taken to attain the defined torque increased when the defined torque value is increased. Figure 10 shows that we found that the master device could also generate a spring-like torque relative to the device’s rotation. At the earliest stage, there was little error, and then the generated torque was almost stable as it increased.

### 4.3. Teleoperation Performance Test for Passive Training 

**Figure 11:**
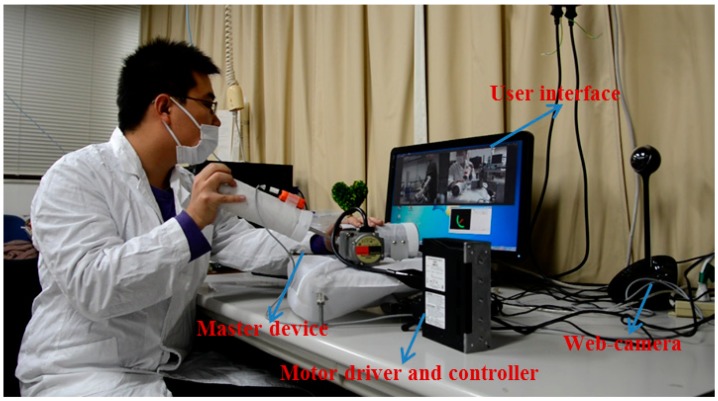
Master side of the implemented telerehabilitation.

**Figure 12:**
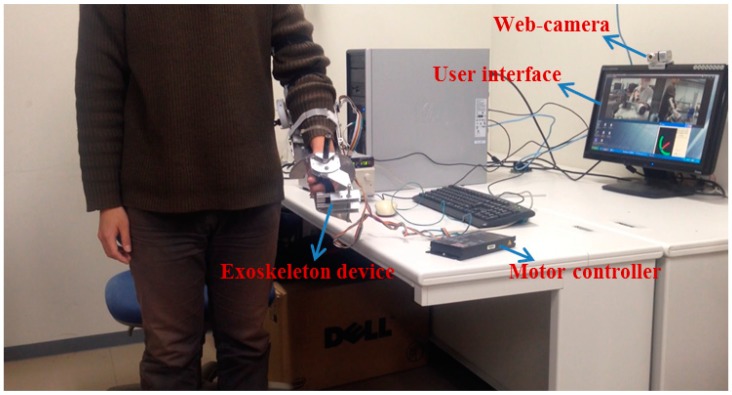
Slave side of the implemented telerehabilitation.

**Figure 13:**
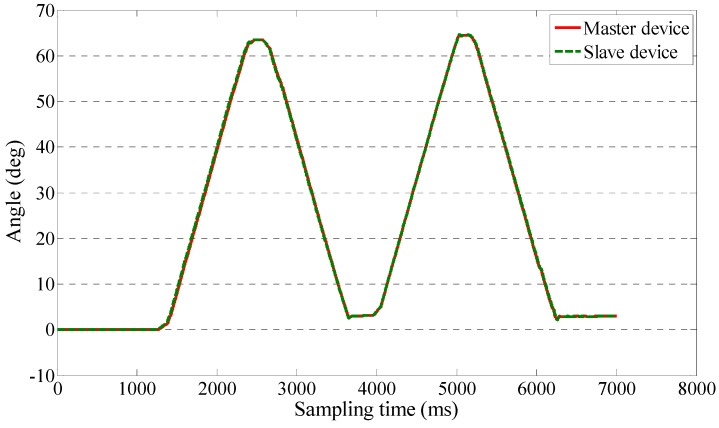
Following of the master device by the exoskeleton device.

**Figure 14:**
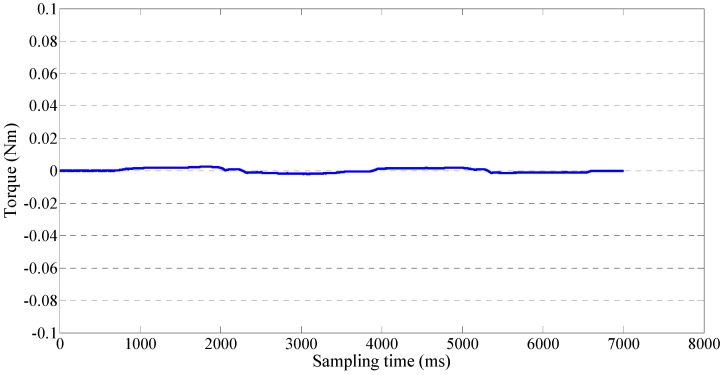
Output torque of the master device.

We implemented a teleoperation to test the slave device can track the motion of therapists while the output impedance of master device is adjusted to near-zero. The master and slave devices were located in different rooms in Kagawa University, where the network latency is not significant. The two sides communicated with transmission control protocol/internet protocol. The experimental setup of the master side is shown in Figure 11. A subject instead of a therapist performed the training as a surrogate therapist. The subject was knowledgeable in rehabilitation training. The user interface included the operation panel, a real-time video, and a virtual model. The operation panel was used for switching the training tasks, saving training data, *etc.* The real-time video was transmitted from the slave side via a web camera. The master system consisted of the motor driver, controller, master device, and an inertia sensor (MTx sensor). The setup of the slave side is shown in Figure 12. We used a healthy subject (age 27 years, male) as a surrogate patient. The design of this telerehabilitation system focuses on elbow joint motor recovery. Therefore, on the slave side, only the motor in the elbow joint was driven and controlled (*i.e.*, the motor installed in the wrist part of the exoskeleton device was not used). After the patient connected his system with the therapist’ side, the therapist started the training while monitoring the status of the patient. In this test, the passive training modality was selected and the output impedance of master device was set to near-zero. In this modality, the patient maintained a relaxed position while the therapist provided the desired motion pattern. The motion from the therapist was measured with the inertia sensor with a 1000-Hz sampling frequency. The exoskeleton device tracked the motion well with a closed-loop position control method. The experimental results are shown in Figure 13 and Figure 14, showing that the exoskeleton device was able to track the motion from therapists well and the output torque from the master device was controlled within a small value. The average time delay during this experiment was 0.6788 ms. The surrogate patient did not feel obvious time delay. As a healthy subject was used as a surrogate patient, the subject was relaxed and followed the motion of the therapists. Therefore, a spasm condition was not tested. The stability of the telrehabilitation system for spastic conditions will be tested in our future studies. 

## 5. Discussion

The inconvenience of visiting rehabilitation centers is expected to create an increasing demand for home-based rehabilitation. These demands could be met by an effective telerehabilitation system that exploits up-to-data internet technology. Traditional telerehabilitation is seldom considered to bring the feeling of real contact between the patient and therapist into the teleoperation. This paper presents a novel telerehabilitation system incorporating a human-upper-limb-like device (master device) for therapists’ use and an exoskeleton device (slave device) for patients’ use. The proposed system provides several solutions to bring the real contact feeling into telerehabilitation. The system can perform both passive and active training. The master device is installed with an SEA that serves two purposes: providing variable impedances while solving the issue of non-backdrivability and accomplishing force detection in a manner similar to a force sensor. As related to force detection, this method measures the position to obtain the force measurement, which is both low cost and low noise.

We tested the constant output impedance and spring-like output impedance by implementing a closed-loop interaction control strategy. In particular, we magnified and detected the deflection of the elastic elements in the master device by a contactless position sensor, which effectively decreased the signal-to-noise ratio. We calculated the relationship between the deflection of the elastic elements and the sensed force (output impedance) with a calibration method. We used a low-noise force sensor during the calibration.

On the patient’s side, an exoskeleton device was used. This easily implemented device is eminently suited to home-based rehabilitation. In particular, if a device is to behave as a surrogate therapist, it must be able to switch between the high joint impedance required for passive training and the near-zero impedance necessary for active training. Patient safety is the most crucial issue during rehabilitation. There are two methods to ensure patient safety in the event of a possible time delay or data loss during the telerehabilitation. The first is to monitor the motor’s current from the exoskeleton device. The motor’s current can reflect the interaction force between the patient and exoskeleton device. The therapist can feel a sudden increase in force by enlarging the deflection of elastic elements when the current is larger than a preset threshold. The second is a torque-limiter mechanism designed in the exoskeleton device.

In the future, the real contact feeling e.g., the force feedback from patients to therapists will be confirmed with more experiments. The stability in e.g., spastic conditions of the telerehabilitation system operated under unexpected time delay will be confirmed as well. The teach-and-replay control method [15] is considered to be used in our system for active training. The passive training requires the motion from therapists can be transmitted to patients in real-time. Therefore, we will improve the stability in future studies.

## 6. Conclusions

We implemented a closed-loop interaction control strategy to control the master device to generate variable impedances. The design mechanism of the master device is compact and low cost after installing a SEA. The actuator also acts as a force-sensing system in place of a traditional force sensor. We calibrated the force-sensing system and found that RMSE (0.004198) was acceptable. Experiments for testing the output impedance of the master device involved constant impedance and spring-like impedance. The time from zero force to the defined force was increased when the defined force is increased during the constant impedance test. During the spring-like impedance test, we found a small instability (error) only at the earliest stage. The web camera transmitted a real-time video between the two sides. The master device was easy to operate and the motion of therapists was transmitted to the slave side while the deflection of elastic elements was adjusted to near-zero to eliminate the non-backdrivable influence. The exoskeleton device was easy to wear and it could track the motion of therapists well.
